# Effectiveness of the non-pharmaceutical public health interventions against COVID-19; a protocol of a systematic review and realist review

**DOI:** 10.1371/journal.pone.0239554

**Published:** 2020-09-29

**Authors:** Shabnam Iezadi, Saber Azami-Aghdash, Akbar Ghiasi, Aziz Rezapour, Hamid Pourasghari, Fariba Pashazadeh, Kamal Gholipour

**Affiliations:** 1 Hospital Management Research Center, Iran University of Medical Sciences, Tehran, Iran; 2 Tabriz Health Services Management Research Center, Tabriz University of Medical Sciences, Tabriz, Iran; 3 HEB School of Business & Administration, University of the Incarnate Word, San Antonio, Texas, United States of America; 4 Health Management and Economics Research Center, Iran University of Medical Sciences, Tehran, Iran; 5 Research Center of Evidence-Based Medicine (EBM), Tabriz University of Medical Sciences, Tabriz, Iran; 6 Social Determinants of Health Research Center, Tabriz University of Medical Sciences, Tabriz, Iran; Chinese Academy of Medical Sciences and Peking Union Medical College, CHINA

## Abstract

**Background:**

Without any pharmaceutical intervention and vaccination, the only way to combat Coronavirus Disease 2019 (COVID-19) is to slow down the spread of the disease by adopting non-pharmaceutical public health interventions (PHIs). Patient isolation, lockdown, quarantine, social distancing, changes in health care provision, and mass screening are the most common non-pharmaceutical PHIs to cope with the epidemic. However, there is neither systematic evidence on the effectiveness of non-pharmaceutical PHIs in controlling the COVID-19 nor on how these interventions work in different contexts. Therefore, in this study we will address two main objectives: 1) to assess the effectiveness of the non-pharmaceutical PHIs in controlling the spread of COVID-19 using a systematic review and meta-analyses; 2) to explore why, how, and for whom these interventions work using a realist review.

**Materials and methods:**

This review study has two main phases. In the first phase of this study, we will extract data from two main types of studies including quasi-experimental studies (such as quasi-randomized trials, controlled before-after studies (CBAs) and interrupted time series studies (ITSs)) and observational studies (such as cohort, case-control, and cross-sectional studies), written in the English language. We will explore effectiveness of the non-pharmaceutical PHIs targeted either suppression or mitigation strategies (or a combination of both) in controlling the COVID-19 epidemics in the community level. Effectiveness will be considered as the changes in mortality rate, incidence rate, basic reproduction number rate, morbidity rate, rates of hospitalization, rates of intensive care unit (ICU) hospitalization, and other health outcomes where possible. We will perform random-effects meta-analyses, if possible, using CMA software. In the second phase, we will conduct a realist review to find out how, why, for whom, and in what circumstances the non-pharmaceutical PHIs work. At the realist review, we will identify and explore Context-Mechanism-Outcome configurations to provide a robust explanation on the effectiveness of the interventions in different contexts using Pawson's 5-step realist review template including: "clarify scope; search for evidence; appraise primary studies and extract data; synthesize evidence and draw conclusions; and disseminate, implement and evaluate". Although the steps are presented in a linear manner, in practice, we will follow them in iterative stages to fill any potential overlap.

**Discussion:**

The findings of this research will provide a crucial insight into how and in which context the non-pharmaceutical PHIs work in controlling the spread of COVID-19. Conducting a systematic review and meta-analysis in line with a realist review will allow us to draw a robust conclusion on the effects and the way in which the interventions work. Understanding the role of contextual factors in the effectiveness of non-pharmaceutical PHIs and the mechanism of this process could enable policymakers to implement appropriate policies and manage the COVID-19 epidemics more efficiently.

**Systematic review registration:**

CRD42020186855.

## Background

Since the beginning of the severe acute respiratory syndrome coronavirus 2 (SARS-CoV-2) outbreak in December 2019 in Wuhan- China and rapid spread all over the world [1], there have been more than 4,500,000 confirmed cases and over 400,000 death by May 16, 2020. On March 11, 2020, World Health Organization (WHO) declared SARS-CoV-2 outbreak as a global pandemic [2]. Coronavirus Disease 2019 (COVID-19) is an infectious disease caused by SARS-CoV-2. Currently, not only no effective immunization is available but also the risk of the infection for the second or third times seems to be possible [2]. Additionally, there is no certain treatment for COVID-19 and most of the available treatments are provided to control the symptoms [3]. Currently, it seems that the best and most effective way to reduce the number of infected cases, as well as the number of COVID-19 related death, is to follow official infection control measures and practice social distancing [2, 4].

COVID-19 disease burden has imposed an overwhelming pressure on health resources and outcomes and economy of both High-Income Countries (HICs) and Low- and Middle-Income Countries (LMICs) [5–7]. Without any pharmaceutical intervention, the only way to slow down the spread of COVID-19 is reducing the interactions among the population [8]. In the early days of the outbreak, some countries implemented a containment strategy [9]. However, the fast spread of the infection in those countries showed that this strategy is not very effective [10]. Therefore, countries adopted more aggressive strategies such as mitigation and suppression strategies. Some examples of such public health strategies include social distancing (also known as physical distancing), contact tracing, travel restrictions, mass screening, and lockdown [11, 12]. Whereas the main goal of mitigation strategies is to slow down the spread of the disease in order to reduce the burden by keeping the peak of hospitalizations (and potential deaths) lower and more spread out so that resources aren’t overwhelmed, the main goal of suppression strategies is to stop the spread of the virus within the community [13]. Suppression is more forceful than mitigation and seeks to flatten the epidemic's curve more rapidly [14]. Three main aims of both mitigation and suppression strategies include 1) flatten the infection curve, 2) reduce the burden on hospitals/infrastructure, and 3) minimize the number of cases and ultimately the number of deaths [15]. Effectiveness of such strategies could be described in relation to the number of new cases detected, change in prevalence or cumulative incidence of the disease, and rate of hospitalizations.

A various number of studies have been conducted to assess the effectiveness of different public health strategies/interventions. For instance, a study by Marc Saez and colleagues (2020) showed how a national lockdown helped to flatten the epidemic curve of COVID-19 [13]. Another study in China, regarding the effectiveness of quarantine and isolation, showed that these interventions may need a longer period to be very effective [16]. Despite these studies, it is not systematically investigated if these interventions have been effective in controlling the spread of the disease and why such interventions work in some regions and not in other regions. Published articles have targeted only a specific aspect of the interventions. For example, one study only compared the effectiveness of medical masks versus N95 respirators [17]. In another study, the researchers only assessed the effectiveness of quarantine among high-risk individuals [18]. Although these studies are very useful in determining the effectiveness of a specific intervention in controlling COVID-19 spread, they cannot explain the variation in success of those strategies in different economic, cultural, or regional conditions. Moreover, available studies have not comprehensively investigated the effectiveness of all implemented public health interventions (PHIs). It is widely accepted that the success of a PHI is related to the contextual factors [19, 20]. Understanding the impact of context is more important due to the current situation and ongoing efforts to reduce the spread of COVID-19 by implementing PHIs [8]. For example, during the 2003 Severe Acute Respiratory Syndrome Coronavirus (SARS) outbreak in Singapore numerous non-pharmaceutical PHIs were implemented effectively, including effective triage and infection control measures in health-care facilities, patient isolation, contact tracing, and universal screening of school-aged children for fever [21]. Nevertheless, the intensity of the impact of these measures was low in comparison to those that have been adopted in China against COVID-19 [22, 23]. Differences in contextual factors such as demography, social and political structure, and health systems' readiness to deliver high-quality services determine the effects of the non-pharmaceutical PHIs on the transmission of COVID-19 [24]. However, there is neither systematic evidence on the effectiveness of non-pharmaceutical PHIs in controlling the COVID-19 nor on how these interventions work in different contexts. Therefore, in this study we will address two main objectives: 1) to assess the effectiveness of the non-pharmaceutical PHIs in controlling the spread of COVID-19 using a systematic review and meta-analyses; 2) to explore why, how, and for whom these interventions work using a realist review.

### Context in PHIs

There is a complexity with public health policies and interventions [25, 26], since their effectiveness depends upon both the unique characteristics of interventions as well as the implementation context. Furthermore, interventions are adaptive, and they may change while implementing in different contexts. The theories related to the implementation of PHIs pinpointed the importance of contextual factors [27]. According to these theories, intervention, implementation, and context are extremely interconnected and influence one another [25]. However, it is not easy to distinguish the boundaries of an intervention from its context, because the boundaries are somewhat haphazard and there are necessarily long-lasting interactions between them. Therefore, implementation of interventions within dynamic contexts makes those boundaries difficult to be recognized [28]. This interaction between intervention and context makes it difficult to discern between items belonging specifically to the intervention and items belonging to the context. This differentiation is necessary for some reasons. First, because of the dynamic nature of adapting an intervention, any changes should be taken into account when evaluating them in a long period of implementation. Second, the interaction between intervention and context is a determinant of the intervention’s effectiveness [29]. In fact, evaluation should be done to determine the interaction between the intervention items and context items and to explore how they impact one another to create perceived outcomes [28].

There are some examples of infectious disease epidemics that highlight the importance of the role of context in the effectiveness of a PHI. For example, during the Zika outbreak in the United States (US) and Brazil, the US experienced colossal political and economic challenges, where Brazil, which had the highest number of Zika cases, experienced higher economic disparity and serious shortages in sanitation and health care capacity. Using a social-ecological framework, Linde-Arias et al. showed that interactions of macro, meso, and micro-level elements determine the reaction of the population to Zika. They also concluded that lack of trust in governments made women more vulnerable because they did not completely follow the health authorities' instructions. This example indicates the role of political and social context in the success of a PHI [30]. Effectiveness of a non-pharmaceutical PHI is tailored to changing the behavior of the target population [31, 32]. That is, if people change their behavior in a way to follow preventive measures in COVID-19 pandemics, the spread of the disease will slow down; and at this regard, there are some theories that explain the behavior change mechanism in the real world.

### Why a realist review of context, and what is the contribution of this study?

Realist review is a robust review and synthesis method that allows reviewers to focus beyond the randomized controlled trials (RCTs) as the basic sources of review documentation, by synthesizing a variety of evidence types [33]. Addressing “how” and “why” questions is the focus of a realist method, especially when exploring the outcomes of the PHIs [27]. The realist review is a method of systematically synthesizing relevant evidence with the aim of the development and refinement of theory by taking both context and outcomes into consideration. Opposed to the conventional systematic review, in the realist review, studies are synthesized in an explanatory way rather than final judgment [27, 33].

The main role of the PHIs in regional and global levels is to control the infections such as Ebola, SARS, Middle East respiratory syndrome coronavirus (MERS), and now COVID-19. These epidemics or pandemics not only are associated with negative health outcomes in a community but result in considerable economic losses as well. Patient isolation, quarantine, lockdown, social distancing, changes in health-care provision (e.g. prioritizing patients for receiving healthcare services in hospitals), mass screening, and vaccination are the most common public health initiatives to control infectious diseases like COVID-19 [34, 35]. In the absence of vaccination and effective drugs, non-pharmaceutical PHIs are being considered as the only way to control COVID-19. Although many countries like the UK and the US have implemented multiple interventions to control the new epidemic [36, 37], there is no doubt that the political, social, and geographical context has affected the success of any [38, 39]. Nevertheless, it is not completely known which intervention(s), why, where, for whom, and how has been effective in slowing down the spread of COVID-19. Therefore, it is essentially important to address the effectiveness of an intervention in its implementation context in order to reduce the current uncertainty around public health initiatives.

Because of the complexity and dynamic nature of the implementation context, results of the same public health measures differ when adopted in various settings. Therefore, it is highly important to take the context into account when exploring the effectiveness of the interventions [40]. Moreover, as mentioned above, context is often missing from studies on the effectiveness of non-pharmaceutical PHIs against COVID-19. Using realist review we will show the interactions between non-pharmaceutical PHIs, characteristics of context, and principal mechanisms of change in controlling the spread of COVID-19. This realist review will answer the questions including what non-pharmaceutical intervention/s work in controlling the spread of COVID-19, in what circumstances, and for which group of population.

## Materials and methods

This review study has two main phases. In the first phase, which is the quantitative part of the study, we will conduct a systematic review to retrieve the robust, relevant studies on effects of non-pharmaceutical PHIs on controlling the COVID-19 epidemics and will explore the scale of the effects. In the second phase, which is the qualitative part, we will conduct a realist review to find out how, why, for whom, and in what circumstances the interventions work in different contexts.

### Phase 1: Systematic review on effectiveness of non-pharmaceutical PHIs

The protocol of the systematic review adheres to the guidance provided in the Preferred Reporting Items for Systematic Reviews and Meta-Analyses Protocols (PRISMA-P) statement, which is filled and provided as supporting information (S1 File). The systematic review will be conducted from September to December 2020. Preferred Reporting Items for Systematic Reviews and Meta-Analyses (PRISMA) statement will be followed to design and outline the report [41].

#### Eligibility criteria for inclusion of studies

*Population*. Target population will be the community population of the countries in which non-pharmaceutical PHIs have been implemented to control and/or manage the COVID-19 during the pandemic.

*Intervention*. Non-pharmaceutical PHIs targeted either suppression or mitigation strategies (or a combination of both) such as patient isolation, social distancing, quarantine, closing educational institutions, lockdown, border restrictions, mass screening, contact tracing, using personal protecting materials, and other similar interventions will be considered as interventions of interest.

*Comparator*. We will have two types of comparators including:1) community population before being involved in non-pharmaceutical PHIs, and 2) community population in which no non-pharmaceutical PHIs have been implemented and only routine care has been provided for infected cases.

*Outcomes*. Effectiveness of non-pharmaceutical PHIs will be assessed as desirable outcomes. Effectiveness will be considered as the changes in mortality rate (confirmed COVID-19 deaths in 100,000 population), incident rate (newly found cases per 100,000 population), morbidity rate (confirmed COVID-19 cases per 100,000 population), and hospitalization rate (COVID-19 hospitalization per 100,000 population). Intensive care unit (ICU) hospitalization rate (COVID-19 ICU hospitalization per COVID-19 hospitalization) and basic reproduction number rate, and other health outcomes (based on the available data) will be considered as additional outcomes.

*Types of studies*. Two main types of studies including quasi-experimental studies (such as quasi-randomized trials, controlled before-after studies (CBAs), and interrupted time series studies (ITSs)) and observational studies (such as cohort, case-control, and cross-sectional studies) written in the English language will be included in the review.

#### Search strategy

Keywords were identified in three key domains including non-pharmaceutical interventions, coronavirus infectious disease, and effectiveness, using initial literature reviews, the opinions of two experts on the subject, and the Medical Subject Heading (MeSH) (Table 1). The search strategy was designed by a well-experienced medical librarian and will be applied to databases. Pilot searches were conducted for each domain separately and for a combination of all domains to make certain that the final search strategy was correctly adapted. A sample search strategy for PubMed is available as supporting information (S2 File). Full search strategies will be provided in the complete review.

**Table 1:** Primary search strategy/search concept grid for the systematic review.

Concept 1 Non-pharmaceutical public health interventions	Concept 2 effectiveness	Concept 3 Coronavirus disease 2019	
patient isolation OR media report	Effectiveness	2019 novel coronavirus disease	
quarantine OR social shield	Efficacy OR treatment outcome	covid-19	
social distance OR physical distance	patient-relevant outcome	sars-cov-2	
travel ban OR travel restriction	health outcome	2019 novel coronavirus infection	
limit travel OR lockdown	new cases	2019-ncov infection	
Stay at home OR Avoid crowd	Incidence OR prevalence	coronavirus disease 2019	**OR**
contact tracing OR partner notification	Hospitalization	coronavirus disease-19	
personal protecting material OR mask	Reproduction number	2019-ncov disease	
suppression OR mitigation	Mortality OR morbidity		
non-contact greetings			
**AND**			

#### Information sources

Major electronic databases will be searched (from December 2019 onwards) including, PubMed, Cochrane Library, Scopus, CINAHL, ProQuest, Embase, and EconLit. Also, reference and citation lists of relevant studies (through Google Scholar citations), relevant reviews, clinical trial registers (Clinical trials.gov), gray literature (Gray.net), preprint databases (such as medRxiv, bioRxiv, and arxiv.org.), the website of WHO, and other relevant evidence will be reviewed carefully.

#### Data extraction

The search results will be imported in the Endnote program to manage the studies and remove the duplicates. After pilot-test of the 10 percent of the studies by two of the authors, the two authors will independently screen the title/abstracts carefully to retrieve eligible studies. A data extraction form was designed based on the study of Tsou and colleagues [42]. The designed extraction table contains the following items: bibliographic information, methods, results, and discussion (S3 File). Three articles' data will be extracted for testing the validity of the data-extraction form. Afterward, the required information from each paper will be extracted by two investigators, separately. In each stage of screening and data extraction, disagreements will be solved by the decision of a third investigator. The PRISMA flow diagram [41] will be used to report the screening and selection results.

#### Critical appraisal of individual sources of evidence

We will carefully and accurately appraise the quality of eligible studies to assure the internal validity of the review results. In order to explore the methodological quality and the risk of bias of the primary research papers, two types of standardized quality assessment tools will be used based on the designs of the studies. Cochrane Effective Practice and Organization of Practice (EPOC) Risk of Bias Tool [43] will be used for appraising the quasi-experimental designs, such as quasi-randomized trials, ITSs, and CBA studies. Suggested risk of bias criteria for studies with a separate control group include: "random sequence generation; allocation concealment; baseline outcome measurements similar; baseline characteristics similar, incomplete outcome data; knowledge of the allocated interventions adequately prevented during the study; protection against contamination; selective outcome reporting; and other risks of bias". We will rate each item as low-risk, high-risk, and unclear-risk [44]. Effective Public Health Practice Project (EPHPP) Quality Assessment Tool for Quantitative Studies will be used for observational studies including cohort, case control, and cross-sectional studies [45]. Previous studies have proved the content and construct validity of the EPHPP [46] and have reported fair interrater agreement for individual domains and excellent agreement for the final grade [47]. The EPHPP contains 20 items in eight domains including "selection bias, study design, confounders, blinding, data collection method, withdrawals and drop-outs, intervention integrity, and analyses". Raters will rate the individual domains and the composite domain as strong, moderate, or weak [45]. Two of the authors will independently appraise each eligible paper. Inter-rater agreement will be calculated by the intraclass correlation coefficient (ICC). Disagreements among the raters will be solved by discussing the third author.

#### Data synthesis

*Descriptive synthesis*. A narrative explanation of the PICO and brief results of each included paper will be arranged in a summary table and will be categorized into bibliographic information, intervention, outcome measures, and main results. A second-tier explanation of outcome measures will then be provided by details and validity of measures will be examined. Considering each study's perspective (health system or societal perspectives), frequencies will be reported for each outcome measure to find any existing pattern in relation to the context of using the measures.

*Quantitative synthesis.* Information extracted from each manuscript will be categorized into the data extraction sheet to demonstrate the whole description of the included studies. Due to the different interventions that we expect to include in the study, we will conduct analysis separately for each type of intervention. Data points from primary observational studies will be used to perform random-effects meta-analyses using CMA:2 (Comprehensive Meta-Analysis) software. To explore the heterogeneity as a priori to the meta-analyses, summary estimates will be predicted. For example, mean differences, standard mean differences, and 95% confidence interval, using the Hartung-Knapp-Sidik-Jonkman random-effects model [48]. Using Forest plots, the extent of heterogeneity among studies will be visualized. Variance between studies by I2 statistics (proportion of variation in prevalence estimates) will be computed to quantify statistical heterogeneity (I2 ranges between 0 and 100%with values of 0–25% demonstrate low heterogeneity, and 75–100% illustrate considerable heterogeneity). If I2 is greater than 50%, the result of the study is considered as heterogeneous and the random effect model will be used for analysis. The results of Tau2 and Cochran Q test will be reported to indicate heterogeneity, in which estimate results with P-value of < 0.05 will be considered statistically significant. Funnel plot will be used to evaluate the possibility of publication bias.

Contingent on the number of eligible studies, we may further stratify the studies based on design; and, where feasible, we will report the association measures including risk ratio, odds ratio, prevalence ratio, hazard ratio, and incidence rate ratio. Furthermore, meta-analyses of adjusted versus non-adjusted measures will be performed separately, if possible. Depending on the data availability, subgroup analyses will be performed by country, gender, comorbidities, and other characteristics. We might perform sensitivity analysis to examine if there would be differences in the synthesis results when all eligible studies are included compared to when only high-quality once are included, and also, to examine that if the results would vary when both published and unpublished studies are included compared to when only the published studies are included.

#### Meta-biases

Using suitable strategies, we will minimize the potential biases in the review process. Unpublished papers including the preprints as well as the gray literature will be included in the review. The potential publication biases will be explored by detecting and assessing the magnitude of reporting biases using a Funnel plot. Furthermore, the quality of the methodology of the systematic review will be assessed by the Assess the Methodology Quality of Systematic Reviews (AMSTAR) tool [49].

### Phase 2: Realist review of the effectiveness of non-pharmaceutical PHIs

Given the complicated, dynamic, and multi-dimensional nature of non-pharmaceutical PHIs [50] and lack of evidence on their effectiveness, we will perform a realist review to qualitatively synthesize the relevant evidence in an explanatory way in order to indicate the mechanisms by which these interventions succeed or fail.

**Fig 1:**
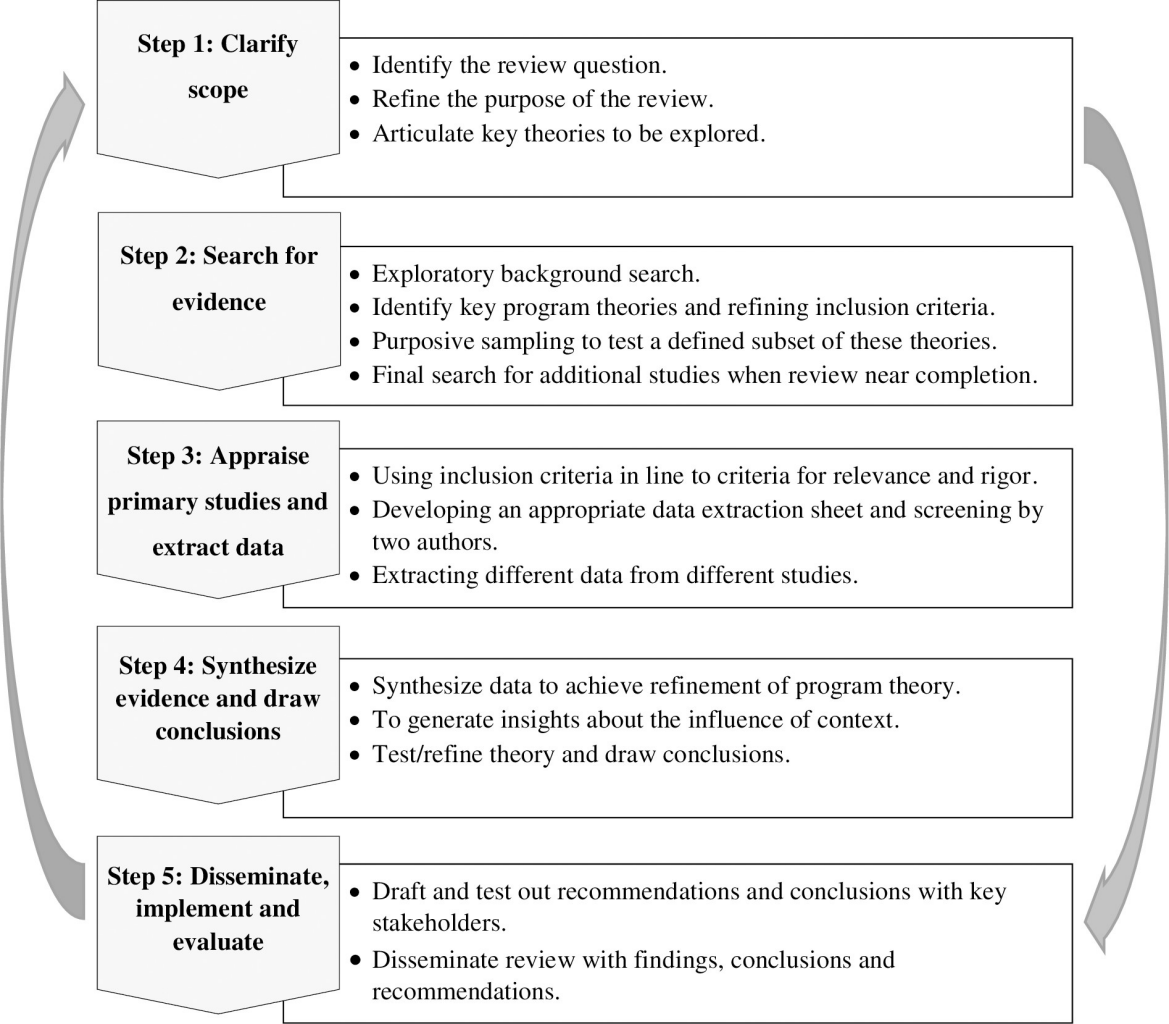
Key steps in the realist review.

Understanding which non-pharmaceutical PHI works successfully requires us to make theoretically causal relationships [27]. Through the course of the realist review, hypotheses of interventions are considered as ideas, beliefs, and desirable outcomes that define how those interventions are supposed to work in the real world. When thinking realistically, programs are considered as theories, and in realist reviews, the theories are explained by intertwined concepts of ‘Context’ (C), ‘Mechanism’ (M), and ‘Outcome’ (O) which are formulated as "C + M = O". To evaluate the effectiveness of the interventions, we will explore their underlying mechanisms and their economic, social, and geographical context to test CMO configurations. Mechanisms are the reactions caused by the context, which either deliberately or haphazardly determine outcomes. Mechanisms could result in different outcomes in different contexts. Each theory could encompasses a variety of CMO configurations, and in this realist review, we will identify and explore CMO configurations to provide a robust explanation on the effectiveness of the interventions in different contexts using Pawson's 5-step realist review template [27]. This template includes "clarify scope; search for evidence; appraise primary studies and extract data; synthesize evidence and draw conclusions; and disseminate, implement and evaluate". Although these steps are presented in a linear way, in practice, we will follow them in iterative stages to fill any potential overlap (Fig 1).

#### Step 1: Clarify scope

Following the guidance of Pawson [27] on realist review, we will begin with clarifying and refining the research question to elicit the basic theories. In realist reviews, in contrast to traditional systematic reviews, because of the dynamic, multifaceted features of the interventions explored, the purpose of the review is explanatory rather than final judgmental [51]. Using a limited number of combinations of keywords related to the implementation context in PHIs, we will conduct comprehensive searches in web-based electronic databases to explore a variety range of key evidence to provide a general view of the topic area.

This initial scoping review of the evidence not only helps the investigators to get a profound comprehension of the research problem but also will facilitate the determination of the relevant ‘program theories’, which in this study is the explanations of how non-pharmaceutical PHIs interact with the implementation context, and how and why these interventions are supposed to succeed. The results of the scoping review at this stage will be a theoretical framework for later steps of the realist review. The primary research question, when conducting the scoping review, is "what non-pharmaceutical PHIs have been adapted to control the COVID-19 epidemics in the community level?". We also will address two sub-questions including: "in what geographical, social, and political contexts have the non-pharmaceutical PHIs been adapted to control the COVID-19 epidemics?"; and, "what outcomes were achieved when the non-pharmaceutical PHIs were adapted to control the COVID-19 epidemics?".

Using the Joanna Briggs Institute (JBI) Manual For Evidence Synthesis [52], we developed inclusion criteria for the scoping review as followings:

*Types of participants*. The target participants in the scoping section is similar to the systematic review and include the community population during the COVID-19 pandemics.
*Concept*. The overarching concept of interest is non-pharmaceutical PHIs that have been used either as suppression or mitigation strategies to control the COVID-19 epidemics.
*Context*. We will focus on community level interventions in both HICs and LMICs.
*Types of evidence sources*. In this stage we will include a wide range of source of studies including observational, quasi-experimental, and epidemiological studies, and reviews.

In order to conduct searches, a three-step search strategy will be used according to the recommendation of JBI [52]. The first step will be a primary limited search of two relevant online databases including PubMed and Scopus. This primary search will be then followed by a screening of the text words included in the title and abstract of the extracted papers as well as the index terms used to characterize the manuscripts. Next, using all identified key terms, a second search will be conducted across all relevant electronic databases for published and unpublished studies (such as gray literature and preprints). Thirdly, the reference and citation lists of the eligible studies will be searched for further sources. Using the eligibility criteria, English language studies published since December 2019 will be retrieved and imported in Endnote and duplicates will be removed. A pilot test of the source selectors will be conducted prior to selecting the eligible studies. Using the eligibility criteria, three of the team members will screen the titles/abstracts of a random sample of 25 studies. Disagreements will be discussed by all team members and modifications will be made to the eligibility criteria where needed. If 75% (or higher) agreement is reached, two reviewers will start screening and extracting eligible papers, independently, and any disagreements will be solved by the decision of a third reviewer. In the scoping review, data charting (data extraction process) will provide a descriptive summary of the findings that fits the questions of the scoping review. A draft charting form was adopted from the JBI methodology guidance for scoping reviews and provided in supporting information (S4 File) [52]. The main information on details and characteristics of the extracted evidence as well as details/results of the evidence will be imported in the form. This might be improved in the review process and the charting form would be updated accordingly. Prior to data charting, the two investigators will pilot-test the form. The final aim of charting the data is to recognize, specify, and summarize the relevant evidence on the topic. The results of the scoping review will be presented in a tabular form as a map of the extracted data. Draft form of the tabular presentation of data for the scoping review is available as supporting information (S5 File). The draft form would be refined and amended during the review process. Finally, a theoretical framework will be developed to clarify the scope of the realist review and outline the later steps of the realist review.

#### Step 2: Search for evidence

There are some differences in searching for relevant evidence between realist reviews and traditional systematic reviews. In contrast to traditional systematic reviews, in which specific types of evidence with specific methodology are selected, in realist review, various sources of relevant information, which cover the topic of the study, make reviewers follow a purposive sampling strategy. Moreover, in realist review, only primary studies, which contribute to the development of a complete picture of reality are included in the synthesis. While other studies with similar results, which do not add any new element, are excluded. By applying multiple search strategies with purposive sampling, reviewers could answer questions or discover specific theories. Reviewers continue to purposively select studies to recognize, test out, or refine the theories in an iterative way until reaching the saturation. As a result, they could guarantee that adequate evidence has been collected to answer the research questions [27, 40].

At this stage, we will carry out systematic searches to find relevant evidence such as process evaluations and qualitative research, to discover and refine the program theories. The search process will be developed gradually to find new and emerging evidence to cover all relevant documents/articles that have addressed the theoretical needs of the research. This process will be iterative and will be continued until reaching saturation, which means that all relevant evidence has been found and included in the review to answer the research questions and refine the theories. All scientific electronic databases and organizational websites will be searched using multiple search strategies, which will be the combinations of the relevant search terms. Furthermore, we will carefully screen the reference and citation lists of relevant studies as well as gray literature to include as many different types of relevant documents as possible in the review. Afterward, we will import all search results into EndNote software to manage them and remove duplicates.

Our inclusion criteria include:

- Nonpharmaceutical programs targeting the control of COVID-19 epidemics in the community.
- Nonpharmaceutical programs targeting community behavior change mechanisms during the COVID-19 epidemics.
- Nonpharmaceutical programs targeting community's behavior during the COVID-19 epidemics.

### Step 3: Appraise primary studies and extract data

At this stage, we will appraise studies based on two main criteria including relevance and rigor. Unlike conventional systematic reviews, criteria of “relevance” for selecting evidence is not about the relevance of the evidence to a specific topic; rather, it is about if the evidence addresses the specific theory, which is under discovery. On the other hand, "rigor" indicates if an explanation concluded by the original researcher is adequately robust to make a methodologically acceptable and trustworthy contribution to the discovery of a specific theory. Although "relevance" and "rigor" are not certain criteria to show the strength of an evidence, they are appropriate to be used as "fitness for purpose criteria" in realist synthesis [27].

Based on the emerging findings and the theory developed in step 1, we will develop ‘Bespoke’ data extraction forms [58] incorporating key elements and research questions. Different from data extraction sheets used in traditional systematic reviews, ‘Bespoke’ data extraction forms will be used mainly to collect information on contextual elements, mechanisms, and outcomes, along with further information on non-pharmaceutical PHIs against COVID-19 pandemics by two independent investigators. Desired outcomes will be changes in the spread of disease and other health outcomes. In addition to information on intervention, contextual elements, mechanisms, and outcomes, we will extract bibliographic information on evidence and strength and limitation of the study, where possible. To appraise the evidence for relevance and rigor, we will use RAMESES (Realist And MEta-narrative Evidence Syntheses: Evolving Standards) Quality Standards for Realist Synthesis [53] applying a ‘fitness for purpose’ approach. Also, we may include the studies with weak methodologies in the synthesis if they contain valuable, relevant information on the research theory. Two reviewers will extract data and categorize them into tables as themes. Interpretation of data will be conducted in a narrative format by judgment and reflection of all team members.

### Step 4: Synthesize evidence and draw conclusions

In contrast to the systematic review and meta-analysis, no quantitative analysis will be conducted at this stage and syntheses of evidence will be conducted narratively. The aim of this step is to discover and refine the research theories to explore the interactions between contexts, mechanisms, and outcomes [54]. Two reviewers will read the included studies carefully and code their contents to identify contexts, mechanisms, outcomes, and CMO configurations. In an iterative way, reviewers will search for repeated patterns of CMOs by comparing the evidence. In the whole process of synthesizing the evidence, the authors will recall the research questions and primary aim of the review in their mind to avoid digressing from the topic. At the end of this stage, reviewers will explain two main issues in a narrative format; first, how, and why non-pharmaceutical PHIs work in controlling the spread of COVID-19 within a specific context. Second, which contextual factors are most important, and how, when and for whom they matter, regarding their impact on the implementation of non-pharmaceutical PHIs.

### Step 5: Disseminate, implement, and evaluate

Finally, findings will be interpreted and presented as evidence-based, applied guidance to be used by a wide range of stakeholders.

## Discussion

The findings of this research will provide a crucial insight into how and in which context the non-pharmaceutical PHIs work in controlling the spread of COVID-19. Understanding the role of contextual factors in the effectiveness of non-pharmaceutical interventions and the mechanism of this process could enable policymakers to implement appropriate policies and manage the epidemics more efficiently.

Countries' different approaches to manage COVID-19 outbreak and remarkably different outcomes indicate the importance of studying the mechanisms in which non-pharmaceutical PHIs work under the effects of contextual factors. Due to the novel and unknown features of the COVID-19, it seems that a set of interventions in each setting need to be used to control the epidemic [18]. Though lockdown, quarantine, contact tracing, and case isolation are proposed as effective interventions to control the epidemic, they may result in different outcomes in different contexts because of the particular features of the COVID-19 such as basic reproduction number, asymptomatic infected cases, and subclinical infection occurrence [55]. On the other hand, contextual factors including economic situations, population density, social norms, social interactions, social dynamics, and perceived risk of the disease, as well as system-level factors such as government financial support and the population's trust in government [56] could influence the effectiveness of both suppression and mitigation strategies.

Comparing the mechanisms and contextual factors in effective management of the outbreak will help policymakers gain more evidence for future pandemics. This information will be important for stakeholders to investigate interventions tailored to the context. A better understanding of non-pharmaceutical PHIs will also inform policymakers about how to develop and implement intervention at a local level. Moreover, conducting a systematic review and meta-analysis in line with a realist review will allow us to draw a robust conclusion on the effects of the interventions and the way in which the interventions work.

We predict some limitations with this study that should be pointed out. First, according to the recent evidence, the same samples of the population have been included in some different publications without providing obvious explanations for the duplicate reporting [57]. To address this issue, we will make a special effort to identify the target population repeatedly reported in different similar studies. Second, we will only include studies in English language, so we will miss a large number of studies in a language other than English. Second, because of the complex nature of the PHIs [50], we will perform meta-analysis separately for each intervention (or a set of similar interventions). This may cause us not to be able to perform meta-analysis for some types of intervention. Based on the guidance produced by the Cochrane Collaboration or the Centre for Reviews and Dissemination (CRD), we planned to conduct narrative synthesis [58] and use Harvest plot method [59] for the condition in which performing the meta-analysis is not possible because of the high heterogeneity of the studies. The Harvest plot is a novel graphical method, which could be used for synthesizing information on PHIs by including all relevant data where meta-analysis is not feasible or sensible [59].

### Dissemination plan

Final reports of this study will be published in peer-reviewed journals as well as the PROSPERO website. All data will be available as supporting information once the review is completed. Also, brief findings will be presented in academic meetings and relevant professional assemblies.

## Supporting information

S1 File PRISMA-P statement. (DOC)

S2 File Sample search strategy for PubMed. (DOCX)

S3 File Data extraction form for systematic review. (DOCX)

S4 File Draft data charting form. (DOCX)

S5 File Draft form of the tabular presentation of data for the scoping review. (DOCX)
