# Supplementary material for: Effectiveness of the non-pharmaceutical public health interventions against COVID-19; a protocol of a systematic review and realist review
Source: PLoS One. 2020 Sep 29;15(9):e0239554. doi: 10.1371/journal.pone.0239554 (PMC7523985; doi:10.1371/journal.pone.0239554)
Supplement: S2 File — (DOCX) [file pone.0239554.s002.docx]

**S2 File. Sample search strategy for PubMed.**

| Search | Query | Results |
| --- | --- | --- |
| #1 | ((((((((2019 novel coronavirus[Title/Abstract]) OR (covid-19[Title/Abstract])) OR (SARS-cov-2 infection[Title/Abstract])) OR ("severe acute respiratory syndrome coronavirus 2"[Title/Abstract])) OR (2019-ncov infection[Title/Abstract])) OR (2019-ncov disease[Title/Abstract])) OR (coronavirus disease 2019[Title/Abstract])) OR (coronavirus disease-19[Title/Abstract])) OR ("2019ncov"[Title/Abstract]) | [39,653](https://pubmed.ncbi.nlm.nih.gov/?term=%28%28%28%28%28%28%28%282019+novel+coronavirus%5BTitle%2FAbstract%5D%29+OR+%28covid-19%5BTitle%2FAbstract%5D%29%29+OR+%28SARS-cov-2+infection%5BTitle%2FAbstract%5D%29%29+OR+%28%22severe+acute+respiratory+syndrome+coronavirus+2%22%5BTitle%2FAbstract%5D%29%29+OR+%282019-ncov+infection%5BTitle%2FAbstract%5D%29%29+OR+%282019-ncov+disease%5BTitle%2FAbstract%5D%29%29+OR+%28coronavirus+disease+2019%5BTitle%2FAbstract%5D%29%29+OR+%28coronavirus+disease-19%5BTitle%2FAbstract%5D%29%29+OR+%28%222019ncov%22%5BTitle%2FAbstract%5D%29&ac=no&sort=relevance) |
| #2 | ("severe acute respiratory syndrome coronavirus 2"[Supplementary Concept]) OR ("covid 19"[Supplementary Concept]) | [18,104](https://pubmed.ncbi.nlm.nih.gov/?term=%28%22severe+acute+respiratory+syndrome+coronavirus+2%22%5BSupplementary+Concept%5D%29+OR+%28%22covid+19%22%5BSupplementary+Concept%5D%29&ac=no&sort=relevance) |
| #3 | #1 OR #2 | [40,951](https://pubmed.ncbi.nlm.nih.gov/?term=%28%28%28%28%28%28%28%28%282019+novel+coronavirus%5BTitle%2FAbstract%5D%29+OR+%28covid-19%5BTitle%2FAbstract%5D%29%29+OR+%28SARS-cov-2+infection%5BTitle%2FAbstract%5D%29%29+OR+%28%22severe+acute+respiratory+syndrome+coronavirus+2%22%5BTitle%2FAbstract%5D%29%29+OR+%282019-ncov+infection%5BTitle%2FAbstract%5D%29%29+OR+%282019-ncov+disease%5BTitle%2FAbstract%5D%29%29+OR+%28coronavirus+disease+2019%5BTitle%2FAbstract%5D%29%29+OR+%28coronavirus+disease-19%5BTitle%2FAbstract%5D%29%29+OR+%28%222019ncov%22%5BTitle%2FAbstract%5D%29%29+OR+%28%28%22severe+acute+respiratory+syndrome+coronavirus+2%22%5BSupplementary+Concept%5D%29+OR+%28%22covid+19%22%5BSupplementary+Concept%5D%29%29&ac=no&sort=relevance) |
| #4 | ((((((((((((((Non-pharmaceutical public health interventions)[Title/Abstract] OR NPIs[Title/Abstract]) OR (Isolat*[Title/Abstract] OR quarantine*[Title/Abstract] OR social shield*[Title/Abstract])) OR (social distanc*[Title/Abstract] OR physical distanc*[Title/Abstract])) OR (Travel* ban*[Title/Abstract] OR travel* restrict*[Title/Abstract] OR Travel* limit*[Title/Abstract])) OR (lockdown[Title/Abstract])) OR ((stay* at home)[Title/Abstract] OR (Stay* home)[Title/Abstract])) OR (avoid*crowded areas[Title/Abstract])) OR (non-contact greet*[Title/Abstract])) OR (contact tracing[Title/Abstract])) OR (partner notification[Title/Abstract])) OR (media* report*[Title/Abstract])) OR (personal protect* material*[Title/Abstract])) OR (mask*[Title/Abstract])) OR (Suppress*[Title/Abstract] OR mitigat*[Title/Abstract]) | [2,799,599](https://pubmed.ncbi.nlm.nih.gov/?term=%28%28%28%28%28%28%28%28%28%28%28%28%28%28Non-pharmaceutical+public+health+interventions%29%5BTitle%2FAbstract%5D+OR+NPIs%5BTitle%2FAbstract%5D%29+OR+%28Isolat%2A%5BTitle%2FAbstract%5D+OR+quarantine%2A%5BTitle%2FAbstract%5D+OR+social+shield%2A%5BTitle%2FAbstract%5D%29%29+OR+%28social+distanc%2A%5BTitle%2FAbstract%5D+OR+physical+distanc%2A%5BTitle%2FAbstract%5D%29%29+OR+%28Travel%2A+ban%2A%5BTitle%2FAbstract%5D+OR+travel%2A+restrict%2A%5BTitle%2FAbstract%5D+OR+Travel%2A+limit%2A%5BTitle%2FAbstract%5D%29%29+OR+%28lockdown%5BTitle%2FAbstract%5D%29%29+OR+%28%28stay%2A+at+home%29%5BTitle%2FAbstract%5D+OR+%28Stay%2A+home%29%5BTitle%2FAbstract%5D%29%29+OR+%28avoid%2Acrowded+areas%5BTitle%2FAbstract%5D%29%29+OR+%28non-contact+greet%2A%5BTitle%2FAbstract%5D%29%29+OR+%28contact+tracing%5BTitle%2FAbstract%5D%29%29+OR+%28partner+notification%5BTitle%2FAbstract%5D%29%29+OR+%28media%2A+report%2A%5BTitle%2FAbstract%5D%29%29+OR+%28personal+protect%2A+material%2A%5BTitle%2FAbstract%5D%29%29+OR+%28mask%2A%5BTitle%2FAbstract%5D%29%29+OR+%28Suppress%2A%5BTitle%2FAbstract%5D+OR+mitigat%2A%5BTitle%2FAbstract%5D%29&ac=no&sort=relevance) |
| #5 | ((("quarantine"[MeSH Terms]) OR (("social distance"[MeSH Terms])) OR ("contact tracing"[MeSH Terms])) OR ("masks"[MeSH Terms]) | [19,448](https://pubmed.ncbi.nlm.nih.gov/?term=%28%28%28%22quarantine%22%5BMeSH+Terms%5D%29+OR+%28%28%22social+distance%22%5BMeSH+Terms%5D%29%29+OR+%28%22contact+tracing%22%5BMeSH+Terms%5D%29%29+OR+%28%22masks%22%5BMeSH+Terms%5D%29&ac=no&sort=relevance) |
| #6 | #4 OR #5 | [2,808,189](https://pubmed.ncbi.nlm.nih.gov/?term=%28%28%28%28%22quarantine%22%5BMeSH+Terms%5D%29+OR+%28%28%22social+distance%22%5BMeSH+Terms%5D%29%29+OR+%28%22contact+tracing%22%5BMeSH+Terms%5D%29%29+OR+%28%22masks%22%5BMeSH+Terms%5D%29%29+OR+%28%28%28%28%28%28%28%28%28%28%28%28%28%28%28Non-pharmaceutical+public+health+interventions%29%5BTitle%2FAbstract%5D+OR+NPIs%5BTitle%2FAbstract%5D%29+OR+%28Isolat%2A%5BTitle%2FAbstract%5D+OR+quarantine%2A%5BTitle%2FAbstract%5D+OR+social+shield%2A%5BTitle%2FAbstract%5D%29%29+OR+%28social+distanc%2A%5BTitle%2FAbstract%5D+OR+physical+distanc%2A%5BTitle%2FAbstract%5D%29%29+OR+%28Travel%2A+ban%2A%5BTitle%2FAbstract%5D+OR+travel%2A+restrict%2A%5BTitle%2FAbstract%5D+OR+Travel%2A+limit%2A%5BTitle%2FAbstract%5D%29%29+OR+%28lockdown%5BTitle%2FAbstract%5D%29%29+OR+%28%28stay%2A+at+home%29%5BTitle%2FAbstract%5D+OR+%28Stay%2A+home%29%5BTitle%2FAbstract%5D%29%29+OR+%28avoid%2Acrowded+areas%5BTitle%2FAbstract%5D%29%29+OR+%28non-contact+greet%2A%5BTitle%2FAbstract%5D%29%29+OR+%28contact+tracing%5BTitle%2FAbstract%5D%29%29+OR+%28partner+notification%5BTitle%2FAbstract%5D%29%29+OR+%28media%2A+report%2A%5BTitle%2FAbstract%5D%29%29+OR+%28personal+protect%2A+material%2A%5BTitle%2FAbstract%5D%29%29+OR+%28mask%2A%5BTitle%2FAbstract%5D%29%29+OR+%28Suppress%2A%5BTitle%2FAbstract%5D+OR+mitigat%2A%5BTitle%2FAbstract%5D%29%29&ac=no&sort=relevance) |
| #7 | #3 AND #6 | [7,200](https://pubmed.ncbi.nlm.nih.gov/?term=%28%28%28%28%28%28%28%28%28%282019+novel+coronavirus%29%5BTitle%2FAbstract%5D+OR+%28covid-19%29%29%5BTitle%2FAbstract%5D+OR+%28SARS-cov-2+infection%29%29%5BTitle%2FAbstract%5D+OR+%282019-ncov+disease%29%29%5BTitle%2FAbstract%5D+OR+%282019-ncov+infection%29%29%5BTitle%2FAbstract%5D+OR+%28coronavirus+disease+2019%29%29%5BTitle%2FAbstract%5D+OR+%28coronavirus+disease-19%29%29%5BTitle%2FAbstract%5D+OR+%28%22severe+acute+respiratory+syndrome+coronavirus+2%22%29%29%5BTitle%2FAbstract%5D+OR+%28%22severe+acute+respiratory+syndrome+coronavirus+2%22%29%29%5BTitle%2FAbstract%5D+OR+%28%222019ncov%22%29%5BTitle%2FAbstract%5D%29+AND+%28%28%28%28%28%28%28%28%28%28%28%28%28%28%28%28%28Non-pharmaceutical+public+health+interventions%29+OR+NPIs%29+OR+%28Isola%2A%5BTitle%2FAbstract%5D+OR+quarantine%2A%5BTitle%2FAbstract%5D+OR+social+shield%2A%5BTitle%2FAbstract%5D%29%29+OR+%28social+distanc%2A%5BTitle%2FAbstract%5D+OR+physical+distanc%2A%5BTitle%2FAbstract%5D%29%29+OR+%28travel+ban%2A%5BTitle%2FAbstract%5D+OR+travel+restriction%2A%5BTitle%2FAbstract%5D%29%29+OR+%28Travel%2A+ban%2A%5BTitle%2FAbstract%5D+OR+travel%2A+restrict%2A%5BTitle%2FAbstract%5D+OR+Travel%2A+limit%2A%5BTitle%2FAbstract%5D%29%29+OR+%28lockdown%5BTitle%2FAbstract%5D%29%29+OR+%28%28stay%2A+at+home%29%5BTitle%2FAbstract%5D+OR+%28Stay%2A+home%29%5BTitle%2FAbstract%5D%29%29+OR+%28avoid%2Acrowded+areas%5BTitle%2FAbstract%5D%29%29+OR+%28non-contact+greet%2A%5BTitle%2FAbstract%5D%29%29+OR+%28contact+tracing%5BTitle%2FAbstract%5D%29%29+OR+%28partner+notification%5BTitle%2FAbstract%5D%29%29+OR+%28media%2A+report%2A%5BTitle%2FAbstract%5D%29%29+OR+%28personal+protect%2A+material%2A%5BTitle%2FAbstract%5D%29%29+OR+%28mask%2A%5BTitle%2FAbstract%5D%29%29+OR+%28Suppress%2A%5BTitle%2FAbstract%5D+OR+mitigat%2A%5BTitle%2FAbstract%5D%29%29&ac=no&sort=relevance) |
| #8 | (((((((((((((((((effective*[Title/Abstract]) OR ("treatment outcome"[MeSH Terms])) OR (patient-relevant outcome*[Title/Abstract])) OR (health Outcome*[Title/Abstract])) OR (treatment efficacy[Title/Abstract])) OR (number of new cases[Title/Abstract])) OR (Incidence[Title/Abstract])) OR ("incidence"[MeSH Terms])) OR (prevalence[Title/Abstract])) OR (Mortality[Title/Abstract])) OR ("mortality"[MeSH Terms])) OR (Morbidity[Title/Abstract])) OR ("morbidity"[MeSH Terms])) OR (basic reproduction number[Title/Abstract])) OR ("basic reproduction number"[MeSH Terms])) OR (Hospitaliz*[Title/Abstract])) OR ("hospitalization"[MeSH Terms])) OR ("prevalence"[MeSH Terms]) | [5,051,679](https://pubmed.ncbi.nlm.nih.gov/?term=%28%28%28%28%28%28%28%28%28%28%28%28%28%28%28%28%28effective%2A%5BTitle%2FAbstract%5D%29+OR+%28%22treatment+outcome%22%5BMeSH+Terms%5D%29%29+OR+%28patient-relevant+outcome%2A%5BTitle%2FAbstract%5D%29%29+OR+%28health+Outcome%2A%5BTitle%2FAbstract%5D%29%29+OR+%28treatment+efficacy%5BTitle%2FAbstract%5D%29%29+OR+%28number+of+new+cases%5BTitle%2FAbstract%5D%29%29+OR+%28Incidence%5BTitle%2FAbstract%5D%29%29+OR+%28%22incidence%22%5BMeSH+Terms%5D%29%29+OR+%28prevalence%5BTitle%2FAbstract%5D%29%29+OR+%28Mortality%5BTitle%2FAbstract%5D%29%29+OR+%28%22mortality%22%5BMeSH+Terms%5D%29%29+OR+%28Morbidity%5BTitle%2FAbstract%5D%29%29+OR+%28%22morbidity%22%5BMeSH+Terms%5D%29%29+OR+%28basic+reproduction+number%5BTitle%2FAbstract%5D%29%29+OR+%28%22basic+reproduction+number%22%5BMeSH+Terms%5D%29%29+OR+%28Hospitaliz%2A%5BTitle%2FAbstract%5D%29%29+OR+%28%22hospitalization%22%5BMeSH+Terms%5D%29%29+OR+%28%22prevalence%22%5BMeSH+Terms%5D%29&ac=no&sort=relevance) |
| #9 | #7 AND #8 | [3,869](https://pubmed.ncbi.nlm.nih.gov/?term=%28%28%28%28%28%28%28%28%28%28%28%28%28%28%28%28%28%28%28%28effective%2A%5BTitle%2FAbstract%5D%29+OR+%28%22treatment+outcome%22%5BMeSH+Terms%5D%29%29+OR+%28patient-relevant+outcome%2A%5BTitle%2FAbstract%5D%29%29+OR+%28health+Outcome%2A%5BTitle%2FAbstract%5D%29%29+OR+%28treatment+efficacy%5BTitle%2FAbstract%5D%29%29+OR+%28number+of+new+cases%5BTitle%2FAbstract%5D%29%29+OR+%28Incidence%5BTitle%2FAbstract%5D%29%29+OR+%28%22incidence%22%5BMeSH+Terms%5D%29%29+OR+%28prevalence%5BTitle%2FAbstract%5D%29%29+OR+%28%22prevalence%22%5BMeSH+Terms%5D%29%29+OR+%28Mortality%5BTitle%2FAbstract%5D%29%29+OR+%28%22mortality%22%5BMeSH+Terms%5D%29%29+OR+%28Morbidity%5BTitle%2FAbstract%5D%29%29+OR+%28%22morbidity%22%5BMeSH+Terms%5D%29%29+OR+%28%22epidemiology%22%5BMeSH+Subheading%5D%29%29+OR+%28%22epidemiology%22%5BMeSH+Subheading%5D%29%29+OR+%28basic+reproduction+number%5BTitle%2FAbstract%5D%29%29+OR+%28%22basic+reproduction+number%22%5BMeSH+Terms%5D%29%29+OR+%28Hospitaliz%2A%5BTitle%2FAbstract%5D%29%29+OR+%28%22hospitalization%22%5BMeSH+Terms%5D%29%29+AND+%28%28%28%28%28%28%28%28%28%28%282019+novel+coronavirus%5BTitle%2FAbstract%5D%29+OR+%28covid-19%5BTitle%2FAbstract%5D%29%29+OR+%28SARS-cov-2+infection%5BTitle%2FAbstract%5D%29%29+OR+%28%22severe+acute+respiratory+syndrome+coronavirus+2%22%5BTitle%2FAbstract%5D%29%29+OR+%282019-ncov+infection%5BTitle%2FAbstract%5D%29%29+OR+%282019-ncov+disease%5BTitle%2FAbstract%5D%29%29+OR+%28coronavirus+disease+2019%5BTitle%2FAbstract%5D%29%29+OR+%28coronavirus+disease-19%5BTitle%2FAbstract%5D%29%29+OR+%28%222019ncov%22%5BTitle%2FAbstract%5D%29%29+OR+%28%28%22severe+acute+respiratory+syndrome+coronavirus+2%22%5BSupplementary+Concept%5D%29+OR+%28%22covid+19%22%5BSupplementary+Concept%5D%29%29%29+AND+%28%28%28%28%28%22quarantine%22%5BMeSH+Terms%5D%29+OR+%28%28%22social+distance%22%5BMeSH+Terms%5D%29%29+OR+%28%22contact+tracing%22%5BMeSH+Terms%5D%29%29+OR+%28%22masks%22%5BMeSH+Terms%5D%29%29+OR+%28%28%28%28%28%28%28%28%28%28%28%28%28%28%28Non-pharmaceutical+public+health+interventions%29%5BTitle%2FA) |
| #10 | (((((((((2019 novel coronavirus)[Title] OR (covid-19))[Title] OR (SARS-cov-2 infection))[Title] OR (2019-ncov disease))[Title] OR (2019-ncov infection))[Title] OR (coronavirus disease 2019))[Title] OR (coronavirus disease-19))[Title] OR ("severe acute respiratory syndrome coronavirus 2"))[Title] OR ("severe acute respiratory syndrome coronavirus 2"))[Title] OR ("2019ncov")[Title] | [42,138](https://pubmed.ncbi.nlm.nih.gov/?term=%28%28%28%28%28%28%28%28%282019+novel+coronavirus%29%5BTitle%5D+OR+%28covid-19%29%29%5BTitle%5D+OR+%28SARS-cov-2+infection%29%29%5BTitle%5D+OR+%282019-ncov+disease%29%29%5BTitle%5D+OR+%282019-ncov+infection%29%29%5BTitle%5D+OR+%28coronavirus+disease+2019%29%29%5BTitle%5D+OR+%28coronavirus+disease-19%29%29%5BTitle%5D+OR+%28%22severe+acute+respiratory+syndrome+coronavirus+2%22%29%29%5BTitle%5D+OR+%28%22severe+acute+respiratory+syndrome+coronavirus+2%22%29%29%5BTitle%5D+OR+%28%222019ncov%22%29%5BTitle%5D&ac=no&sort=relevance) |
| #11 | #8 AND #10 | [8,430](https://pubmed.ncbi.nlm.nih.gov/?term=%28%28%28%28%28%28%28%282019+novel+coronavirus%5BTitle%5D%29+OR+%28covid-19%5BTitle%5D%29%29+OR+%28SARS-cov-2+infection%5BTitle%5D%29%29+OR+%282019-ncov+disease%5BTitle%5D%29%29+OR+%282019-ncov+infection%5BTitle%5D%29%29+OR+%28coronavirus+disease+2019%5BTitle%5D%29%29+OR+%28%22severe+acute+respiratory+syndrome+coronavirus+2%22%5BTitle%5D%29%29+OR+%28%222019ncov%22%5BTitle%5D%29%29+AND+%28%28%28%28%28%28%28%28%28%28%28%28%28%28%28%28%28%28effective%2A%5BTitle%2FAbstract%5D%29+OR+%28%22treatment+outcome%22%5BMeSH+Terms%5D%29%29+OR+%28patient-relevant+outcome%2A%5BTitle%2FAbstract%5D%29%29+OR+%28health+Outcome%2A%5BTitle%2FAbstract%5D%29%29+OR+%28treatment+efficacy%5BTitle%2FAbstract%5D%29%29+OR+%28number+of+new+cases%5BTitle%2FAbstract%5D%29%29+OR+%28Incidence%5BTitle%2FAbstract%5D%29%29+OR+%28%22incidence%22%5BMeSH+Terms%5D%29%29+OR+%28prevalence%5BTitle%2FAbstract%5D%29%29+OR+%28Mortality%5BTitle%2FAbstract%5D%29%29+OR+%28%22mortality%22%5BMeSH+Terms%5D%29%29+OR+%28Morbidity%5BTitle%2FAbstract%5D%29%29+OR+%28%22morbidity%22%5BMeSH+Terms%5D%29%29+OR+%28basic+reproduction+number%5BTitle%2FAbstract%5D%29%29+OR+%28%22basic+reproduction+number%22%5BMeSH+Terms%5D%29%29+OR+%28Hospitaliz%2A%5BTitle%2FAbstract%5D%29%29+OR+%28%22hospitalization%22%5BMeSH+Terms%5D%29%29+OR+%28%22prevalence%22%5BMeSH+Terms%5D%29%29&ac=no&sort=relevance) |
| #12 | #11 AND #6 | [2,130](https://pubmed.ncbi.nlm.nih.gov/?term=%28%28%28%28%28%28%28%28%282019+novel+coronavirus%5BTitle%5D%29+OR+%28covid-19%5BTitle%5D%29%29+OR+%28SARS-cov-2+infection%5BTitle%5D%29%29+OR+%282019-ncov+disease%5BTitle%5D%29%29+OR+%282019-ncov+infection%5BTitle%5D%29%29+OR+%28coronavirus+disease+2019%5BTitle%5D%29%29+OR+%28%22severe+acute+respiratory+syndrome+coronavirus+2%22%5BTitle%5D%29%29+OR+%28%222019ncov%22%5BTitle%5D%29%29+AND+%28%28%28%28%28%28%28%28%28%28%28%28%28%28%28%28%28%28effective%2A%5BTitle%2FAbstract%5D%29+OR+%28%22treatment+outcome%22%5BMeSH+Terms%5D%29%29+OR+%28patient-relevant+outcome%2A%5BTitle%2FAbstract%5D%29%29+OR+%28health+Outcome%2A%5BTitle%2FAbstract%5D%29%29+OR+%28treatment+efficacy%5BTitle%2FAbstract%5D%29%29+OR+%28number+of+new+cases%5BTitle%2FAbstract%5D%29%29+OR+%28Incidence%5BTitle%2FAbstract%5D%29%29+OR+%28%22incidence%22%5BMeSH+Terms%5D%29%29+OR+%28prevalence%5BTitle%2FAbstract%5D%29%29+OR+%28Mortality%5BTitle%2FAbstract%5D%29%29+OR+%28%22mortality%22%5BMeSH+Terms%5D%29%29+OR+%28Morbidity%5BTitle%2FAbstract%5D%29%29+OR+%28%22morbidity%22%5BMeSH+Terms%5D%29%29+OR+%28basic+reproduction+number%5BTitle%2FAbstract%5D%29%29+OR+%28%22basic+reproduction+number%22%5BMeSH+Terms%5D%29%29+OR+%28Hospitaliz%2A%5BTitle%2FAbstract%5D%29%29+OR+%28%22hospitalization%22%5BMeSH+Terms%5D%29%29+OR+%28%22prevalence%22%5BMeSH+Terms%5D%29%29%29+AND+%28%28%28%28%28%22quarantine%22%5BMeSH+Terms%5D%29+OR+%28%28%22social+distance%22%5BMeSH+Terms%5D%29%29+OR+%28%22contact+tracing%22%5BMeSH+Terms%5D%29%29+OR+%28%22masks%22%5BMeSH+Terms%5D%29%29+OR+%28%28%28%28%28%28%28%28%28%28%28%28%28%28%28Non-pharmaceutical+public+health+interventions%29%5BTitle%2FAbstract%5D+OR+NPIs%5BTitle%2FAbstract%5D%29+OR+%28Isolat%2A%5BTitle%2FAbstract%5D+OR+quarantine%2A%5BTitle%2FAbstract%5D+OR+social+shield%2A%5BTitle%2FAbstract%5D%29%29+OR+%28social+distanc%2A%5BTitle%2FAbstract%5D+OR+physical+distanc%2A%5BTitle%2FAbstract%5D%29%29+OR+%28Travel%2A+ban%2A%5BTitle%2FAbstract%5D+OR+travel%2A+restrict%2A%5BTitle%2FAbstract%5D+OR+Travel%2A+limit%2A%5BTitle%2FAbstract%5D%29%29+) |
| #13 | (((((((((((((((Isola*[Title] OR quarantine*[Title] OR social shield*[Title]) OR ((Non-pharmaceutical public health interventions)[Title] OR NPIs[Title])) OR (social distanc*[Title] OR physical distanc*[Title])) OR (Travel* ban*[Title] OR travel* restrict*[Title] OR travel* limit*[Title])) OR (lockdown[Title])) OR ((stay* at home)[Title] OR (Stay* home)[Title])) OR (avoid*crowded areas[Title])) OR (non-contact greet*[Title])) OR (contact tracing[Title])) OR (partner notification[Title])) OR (Media* report*[Title])) OR (personal protect* material*[Title])) OR (mask*[Title])) OR (Suppress*[Title])) OR (mitigat*[Title])) OR ((("masks"[MeSH Terms]) OR ("contact tracing"[MeSH Terms])) OR ("quarantine"[MeSH Terms])) | 531,359 |
| #14 | #8 AND #10 AND #13 | [471](https://pubmed.ncbi.nlm.nih.gov/?term=longquery71555c813487f86f6857&sort=&long_term_hash=longquery71555c813487f86f6857) |
