# Supplementary material for: Effectiveness of the non-pharmaceutical public health interventions against COVID-19; a protocol of a systematic review and realist review
Source: PLoS One. 2020 Sep 29;15(9):e0239554. doi: 10.1371/journal.pone.0239554 (PMC7523985; doi:10.1371/journal.pone.0239554)
Supplement: S3 File — (DOCX) [file pone.0239554.s003.docx]

**S3 File. Data extraction form for systematic review.**

| Data Collection Item | Item description / sub-groups |
| --- | --- |
| Bibiliographic Information | |
| Author |  |
| Title |  |
| Year of publication |  |
| Country |  |
|  | |
| Methods | |
| Intervention |  |
| Aim |  |
| Objectives |  |
| Participant characteristics | Intervention group |
|  | Comparator details |
| Outcome measures | mortality |
|  | incidence |
|  | basic reproduction number |
|  | morbidity |
|  | hospitalization |
|  | intensive care unit (ICU) hospitalization |
|  | other health outcomes (specify) |
|  | |
| Results | |
| Study parameters | The values, ranges, references, and, if used, probability distributions for all parameters. Reasons or sources for distributions used to represent uncertainty where appropriate. |
| outcomes | Mean values for the main categories of estimated outcomes of interest, as well as mean differences between the comparator groups. |
| Characterising heterogeneity | The differences outcomes that can be explained by variations between subgroups of patients with different baseline characteristics or other observed variability in effects that are not reducible by more information. |
|  | |
| Discussion | |
| Study findings, limitations, generalisability, and current knowledge | Summarise key study findings and describe how they support the conclusions reached. Discuss limitations and the generalisability of the findings and how the findings fit with current knowledge. |
