# Supplementary material for: Effectiveness of the non-pharmaceutical public health interventions against COVID-19; a protocol of a systematic review and realist review
Source: PLoS One. 2020 Sep 29;15(9):e0239554. doi: 10.1371/journal.pone.0239554 (PMC7523985; doi:10.1371/journal.pone.0239554)
Supplement: S4 File — (DOCX) [file pone.0239554.s004.docx]

**S4 File. Data extraction table for scoping review**

| **Scoping Review Details** | |
| --- | --- |
| **Review main objective:** to explore non-pharmaceutical PHIs adapted to control the COVID-19 epidemics in the community level |  |
| **Review sub-objectives:**  1- to explore the geographical, social, and political contexts in which the non-pharmaceutical PHIs have been adapted to control the COVID-19 epidemics.  2- to explore the outcomes achieved when the non-pharmaceutical PHIs were adapted to control the COVID-19 epidemics. |  |
| **Inclusion/Exclusion Criteria** | |
| Population |  |
| Concept |  |
| Context |  |
| Types of evidence source |  |
| **Evidence source Details and Characteristics** | |
| Citation details (e.g. author/s, date, title, journal, volume, issue, pages) |  |
| Context/Country |  |
| Participants (details e.g. age/sex and number) |  |
| **Details/Results extracted from source of evidence (in relation to the concept of the scoping review)** | |
| Types of the non-pharmaceutical PHI |  |
| Suppression or mitigation strategy |  |
| Time period |  |
| Outcomes |  |
| Results |  |
