# Supplementary material for: Effectiveness of the non-pharmaceutical public health interventions against COVID-19; a protocol of a systematic review and realist review
Source: PLoS One. 2020 Sep 29;15(9):e0239554. doi: 10.1371/journal.pone.0239554 (PMC7523985; doi:10.1371/journal.pone.0239554)
Supplement: S5 File — (DOCX) [file pone.0239554.s005.docx]

**S5 File. Draft form of the tabular presentation of data for the scoping review**

| **Parameter** | **Results** |
| --- | --- |
| Numbers of publications | - Total number of publications - Number of publications every month |
| Types of studies/evidence | - Quasi-experimental studies  1. quasi-randomized trials 2. controlled before-after studies (CBAs) 3. interrupted time series studies (ITSs)  - Observational studies  1. cohort 2. case-control 3. cross-sectional studies  - Reviews - Case studies/reports - Government documents - Other (specify) |
| Population/s identified | - Population size - Ethnicity - Race - Socioeconomic status - Gender - Age group - Other (specify) |
| Social characteristics of context | - Social norms - Specific cultural features |
| Political characteristics of context | - Political structure - Health system structure - Other (specify) |
| Geographical characteristics of context | - Geographic area (length and breadth of the country) - Climate - population density |
| Non-pharmaceutical PHIs | - Patient isolation - Social distancing - Quarantine - Lockdown - Border restrictions - Mass screening - Contact tracing - Using personal protecting materials - Other (specify) |
| Strategies | - Suppression strategy - Mitigation strategy |
| Outcomes | - Mortality - Incidence - Basic reproduction number - Morbidity - Hospitalization - Hospitalization in intensive care unit (ICU) - Other health outcomes (specify) |
